# Improving Patient to Patient CT Value Uniformity with an Individualized Contrast Medium Protocol Tailored to Body Weight and Contrast Medium Concentration in Coronary CT Angiography

**DOI:** 10.1371/journal.pone.0132412

**Published:** 2015-07-13

**Authors:** Yan Xing, Gulina Azati, Cun-xue Pan, Jun Dang, Sailendra Jha, Wen-ya Liu

**Affiliations:** Department of Radiology, the First Affiliated Hospital of Xinjiang Medical University, Urumqi, Xinjiang, China; Affiliated Hospital of North Sichuan Medical College, CHINA

## Abstract

To determine whether body weight and concentration dependent contrast medium (CM) injection protocols can improve patient to patient CT value uniformity more than the conventional injection protocols with fixed injection parameters in coronary CT angiography (CCTA), one hundred and sixty patients who underwent CCTA were prospectively randomized into two groups. Group A (n = 80) used individualized-protocol with adjusted injection rate based on patient weight and contrast medium concentration to obtain constant iodine load of 280 mgI/kg while group B (n = 80) followed the conventional contrast injection protocol with total injection volume of 80ml and constant injection rate of 5.5ml/s. For both groups, patients were further divided into four subgroups with different CM concentrations: A1, B1 (300 mg I/ml); A2, B2 (320 mg I/ml); A3, B3 (350 mg I/ml) and A4 and B4 (370 mg I/ml). For each patient, the CT values of the ascending aorta, left ventricle and coronary arteries were measured. One-way analysis of variance was used to compare CT values among subgroups. Among the subgroups of A, sufficient attenuation of greater than 300HU was obtained in all target vessels with no difference among them. Among the subgroups of B, the CT values had significant difference in left ventricle, left circumflex branch, proximal and distal segment of the right coronary artery (all p < 0.05), and the attenuation with 300 mg I/ml CM concentration was significantly lower than that with 370 mg I/ml. Compared with group B, group A used less volume (62.83 ml vs. 80.00 ml, P<0.001) and lower rate (5.21 ml/s vs. 5.50 ml/s, P<0.001) of CM. Compared with the conventional contrast medium injection protocol with fixed volume and injection rate, the individualized-protocol based on patient weight and contrast concentration provides overall contrast dose reduction and achieves more homogenous attenuation among different coronary vessels and patients.

## Introduction

Coronary computed tomography angiography (CCTA) has emerged as a useful diagnostic tool for the noninvasive assessment of coronary artery disease[1]. Although CT technology for CCTA has been steadily improving to provide higher diagnostic accuracy, the contrast medium (CM) injection protocol has had little change[2]. In the clinical practice, the CM injection protocol with a fixed infusion speed and volume is still widely used, that may lead to the magnitude of contrast enhancement decrease proportionally with an increase in patient weight. Therefore, when a consistent contrast enhancement is desired, the amount of iodine should be adjusted for the body weight[3].The body weight tailored CM injection protocol during CCTA has been reported in several studies, but the studies have been limited to CM of a certain concentration[4, 5]. Contrast media of various concentrations are used in current clinical practice and may change the degrees of enhancement. How to improve patient to patient uniformity when applying contrast media of various concentrations is still an open question. In this study we proposed an individualized-protocol with adjusted injection rate based on patient weight and contrast medium concentration to obtain constant iodine load of 280 mg I/kg, and to evaluate the effectiveness of this protocol for achieving similar enhancement of coronary arteries among patients using different CM concentrations.

## Materials and Methods

### General data

This prospective study was approved by the Ethics Committee of the first affiliated hospital of Xinjiang medical university and the consent obtained from all participants was written. All relevant data are within the paper (S1 Dataset, S2 Dataset, S3 Dataset and S4 Dataset). This study was conducted from September 2013 to February 2014. One hundred and sixty patients who were assessed with a low-medium risk of coronary artery disease and planned to undergo CCTA were enrolled and randomized into weight-adjusted iodine-load protocol group (Group A, n = 80) and fixed contrast medium volume protocol group (group B, n = 80). The following patients were excluded: patients with a known history of allergy to iodine contrast medium; patients with arrhythmia; patients with renal insufficiency (glomerular filtration rate (GFR) <30 ml/Min); patients diagnosed as having coronary artery disease (with a history of myocardial infarction, coronary angioplasty, coronary stent implantation, or coronary artery bypass grafting). Nine patients with severe coronary calcification and 4 patients with severe coronary stenosis or occlusion, which affected the measurement of coronary CT attenuation, were also excluded. The general data of patients are shown in Table 1; there were no differences in sex, age, height, body weight, and body mass index (BMI) between the two groups (*P*>0.05).

**Table 1:** Baseline data of patients.

Indicator	Group A(*n* = 80)	Group B(*n* = 80)	Statistic	*P*
**Male (%)**	43(53.75)	50(62.50)	1.258	0.262
**Age (year)**	57.06±11.25	56.40±11.58	0.367	0.714
**Height (cm)**	166.25±9.63	168.09±8.12	1.305	0.194
**Body weight(kg)**	75.71±13.04	76.06±11.58	0.179	0.858
**BMI(kg/m** ^**2**^ **)**	27.28±3.44	26.87±3.39	0.747	0.456

### Scanning equipment and parameters

All subjects underwent CT scanning on a 64 row multislice CT (Discovery CT 750HD, GE Healthcare, Milwaukee, WI) using the cardiac scan mode. They had sinus rhythm and oral Betaloc(Betaloc25~50 mg) was used as necessary prior to examination to control the heart rates to<85 bpm before scanning. After breathing training patients were connected to an ECG gating machine. For each patient, localization scan was performed first followed by a plain scan to obtain the calcification score. Contrast enhanced scan from 2cm beneath the carina of trachea to 2cm beneath the diaphragm was performed with retrospective ECG gating, and the delayed scan time was determined based on the low-dose pre-injection time. The other scanning parameters were as follows: tube voltage: 120 kV, tube current: 300~450 mA, gantry rotation time: 0.35 s/r, pitch: 0.16~0.22, collimator width: 64×0.625 mm.

### CM injection protocols

The individualized-protocol group (Group A) was further randomized into 4 subgroups (n = 20 for each subgroup) based on CM concentration.SubgroupA1: 300 mg I/ml(Ultravist, Schering);subgroup A2: 320 mg I/ml(Ioversol, HengRui);subgroup A3: 350 mg I/ml (Omnipaque, Nycomed) and subgroup A4: 370 mg I/ml(Iopamiro, Bracco). The same contrast medium injection time (12s) and constant iodine load (dose/kg) of 280mg I/kg were used for different subgroups. The weight and CM concentration-adjusted total CM volume and injection rate were determined using formula (1) and (2).

$$\text{Contrast medium volume}\;\left(\text{ml}\right)=\;\frac{Weight\;\left(kg\right)\times 280(mgI/kg)}{Contrast\;medium\;concentration\;(mgI/ml)}$$ Formula (1)

$$\text{Injection Speed}\;(\text{ml}/\text{s})\;=\frac{Contrast\;medium\;volume\;(ml)}{12\;s}$$ Formula (2)

We set a limit for the contrast injection speed to less than 6.0 ml/s in this study. In order not to exceed this contrast flow rate limit, for patients in the individualized-protocol group the maximum weights were limited to 78kg, 84kg, 91kg, and 96kg in the 300 mg I/ml, 320 mg I/ml, 350 mg I/ml and 370 mg I/ml contrast concentration subgroups, respectively.

The conventional-protocol group (Group B) was also randomly divided into 4 subgroups (n = 20 for each subgroup) based on CM concentration: 300 mg I/ml (subgroup B1), 320 mg I/ml (subgroup B2), 350 mg I/ml (subgroup B3) and 370 mg I/ml (subgroup B4). A fixed injection protocol with a constant injection speed of 5.5 ml/s and total volume of 80 ml was used for patients in group B.

In both groups, the dual high-pressure syringe double-phase injection technique was used. In the second phase, both groups followed by a flush of 50 ml saline at a same flow rate as the first phase.

### Image analysis

The axial-view images acquired were sent to a GE Advantage Workstation 4.4 (GE Healthcare, Milwaukee, WI). CT attenuation in target vessels for all included subjects was measured by a radiologist with 11-year experience in cardiovascular imaging diagnosis who was blinded to the clinical data of patients and the CM injection protocols. Coronary artery segments were defined based on New York Heart Association (NYHA) coronary segment definition[6]. CT attenuation values of the following vessels were measured: aortic opening (AO) at the level of left coronary artery opening, left ventricle (LV) at the level of bicuspid valve opening, proximal segment of left anterior descending branch (LAD), proximal segment of left circumflex branch (LCX), proximal segment of right coronary artery (RCA), and distal segment of right coronary artery (RCA-PDA) or posterior descending coronary artery (PDA). The measurement was performed at a fixed window width (WW) of 800 HU and a fixed window level(WL) of 100 HU. The region of interest (ROI) for AO and LV had a size of 200 mm^2^, and the ROI for other branches was taken as large as possible avoiding calcification and non-calcification plaques to guarantee the accuracy of CT attenuation measured. Coefficient of variation (CV) of CT attenuation values in target vessels was calculated as CV = (SD÷M) ×100%, where SD and M refer to the standard deviation and mean of the CT attenuation, respectively.

### Statistical analysis

The database was created with Microsoft Excel, and statistical analysis was performed with SPSS 17.0 statistical software package. Continuous variables were presented as mean ± standard deviation (*X*±*S*), while the numeration data were presented as percentage or frequency. If the CT attenuation data obtained with different concentrations of CM in group A and group B had normal distribution and homogeneity of variance, they were analyzed using One-Way ANOVA, and the inter-group comparison was performed with LSD method; if these data had heterogeneity of variance, they were analyzed using Kruskal-Wallis H test, and the inter-group comparison was performed with Tamhane method. α = 0.05 was defined as a level of significance, and P>α suggested no statistically significant difference.

## Results

### Comparison of vessel enhancement degree under individualized-protocol and conventional-protocol.

**Fig 1:**
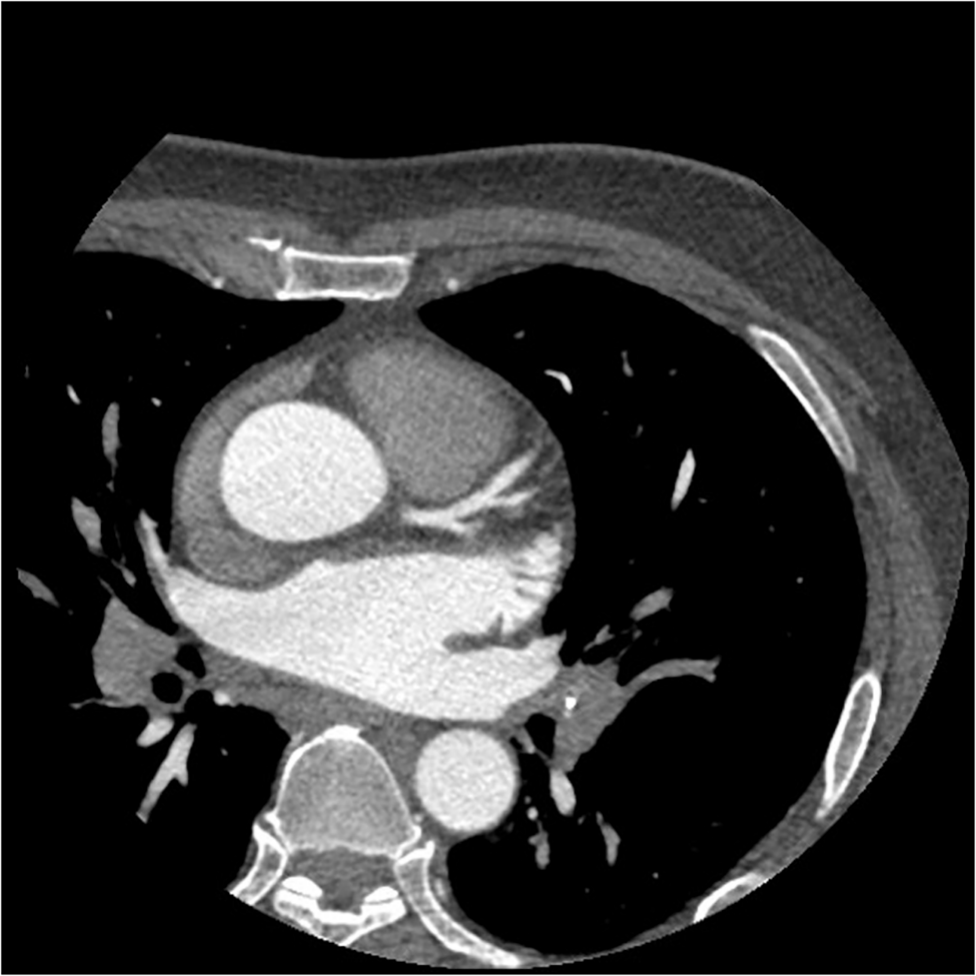
The axial image of CCTA in subgroup A1. Sixty-one years old, female, 65 kg. Injection speed of 5.1 ml/s and injection volume of 61 ml. CT values in target vessels are as following: AO = 414.08HU, LMA = 397.76HU, LAD = 376.52HU, LCX = 358.03HU.

**Fig 2:**
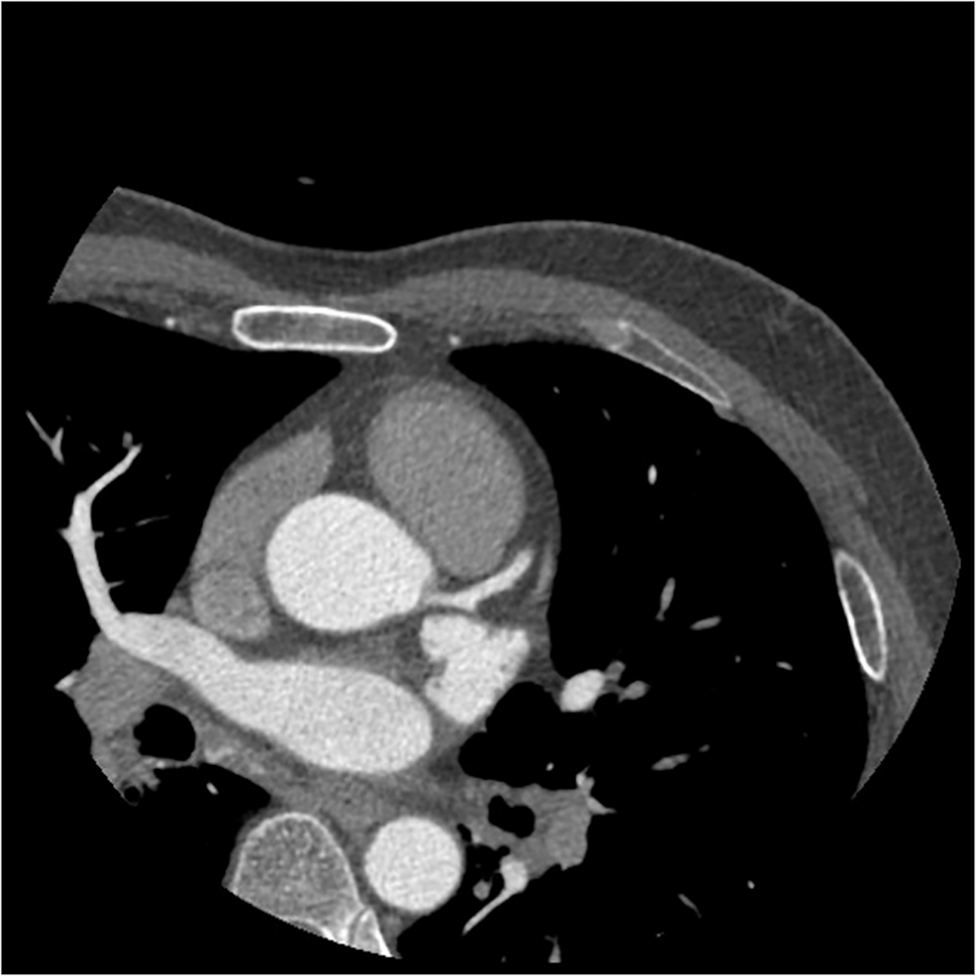
The axial image of CCTA in subgroup A2. Sixty-nine years old, male, 81 kg. Injection speed of 5.9 ml/s and injection volume of 71 ml. CT values in target vessels areas following: AO = 400.24HU, LMA = 389.13HU, LAD = 367.88HU, LCX = 354.84HU.

**Fig 3:**
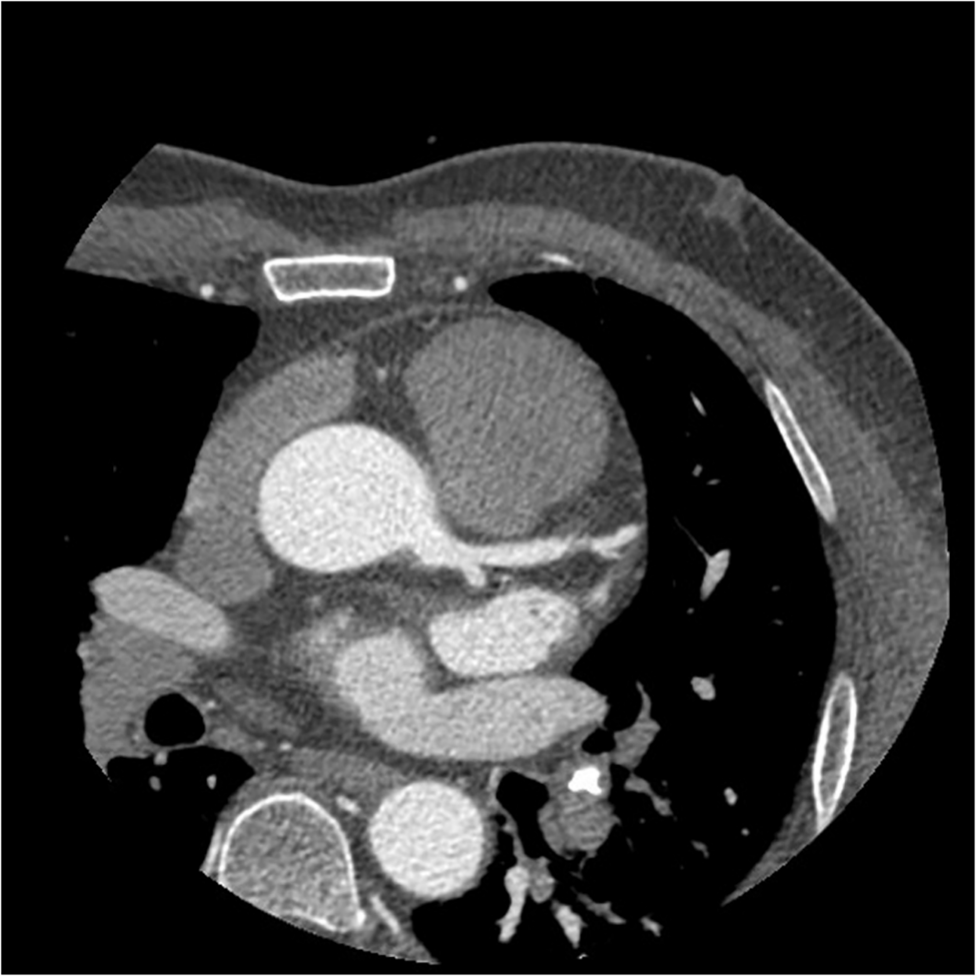
The axial image of CCTA in subgroup A3. Fifty-two years old, male, 90 kg. Injection speed of 6.0 ml/s and injection volume of 72 ml. CT values in target vessels areas following: AO = 410.44HU, LMA = 385.42HU, LAD = 379.86HU, LCX = 349.74HU.

**Fig 4:**
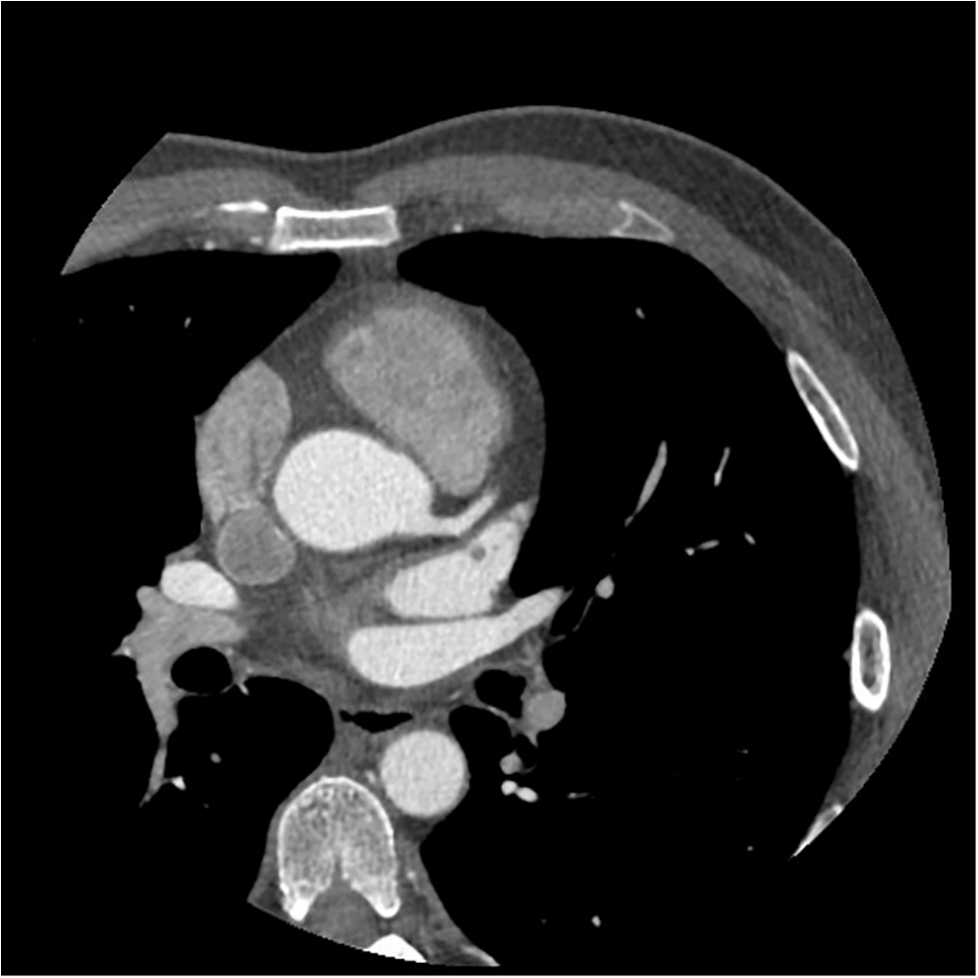
The axial image of CCTA in subgroup A4. Fifty-eight years old, male, 76 kg. Injection speed of 4.8 ml/s and injection volume of 58 ml. CT values in target vessels are as following: AO = 401.62HU, LMA = 391.23HU, LAD = 353.88HU, LCX = 343.98HU.

**Fig 5:**
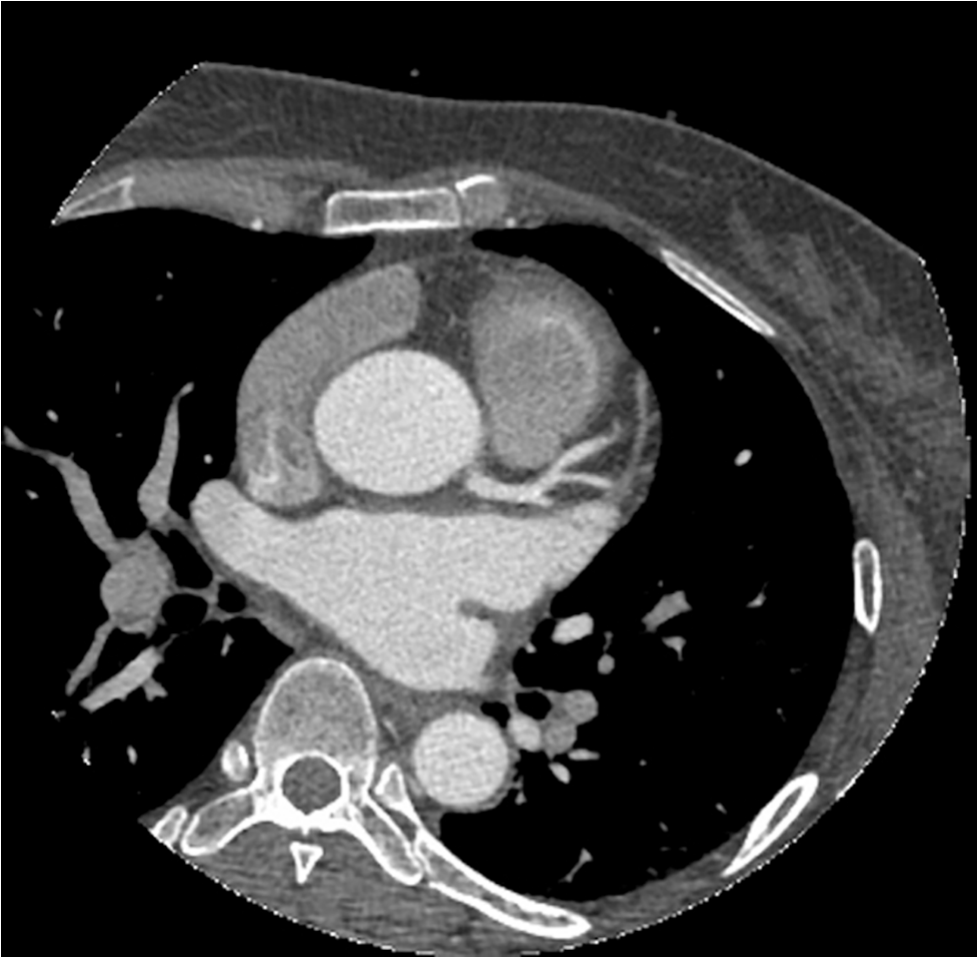
The axial image of CCTA in subgroup B1. Fifty-five years old, male, 76kg. CT values in target vessels are as following: AO = 321.23HU, LMA = 311.59HU, LAD = 298.32HU, LCX = 285.01HU.

**Fig 6:**
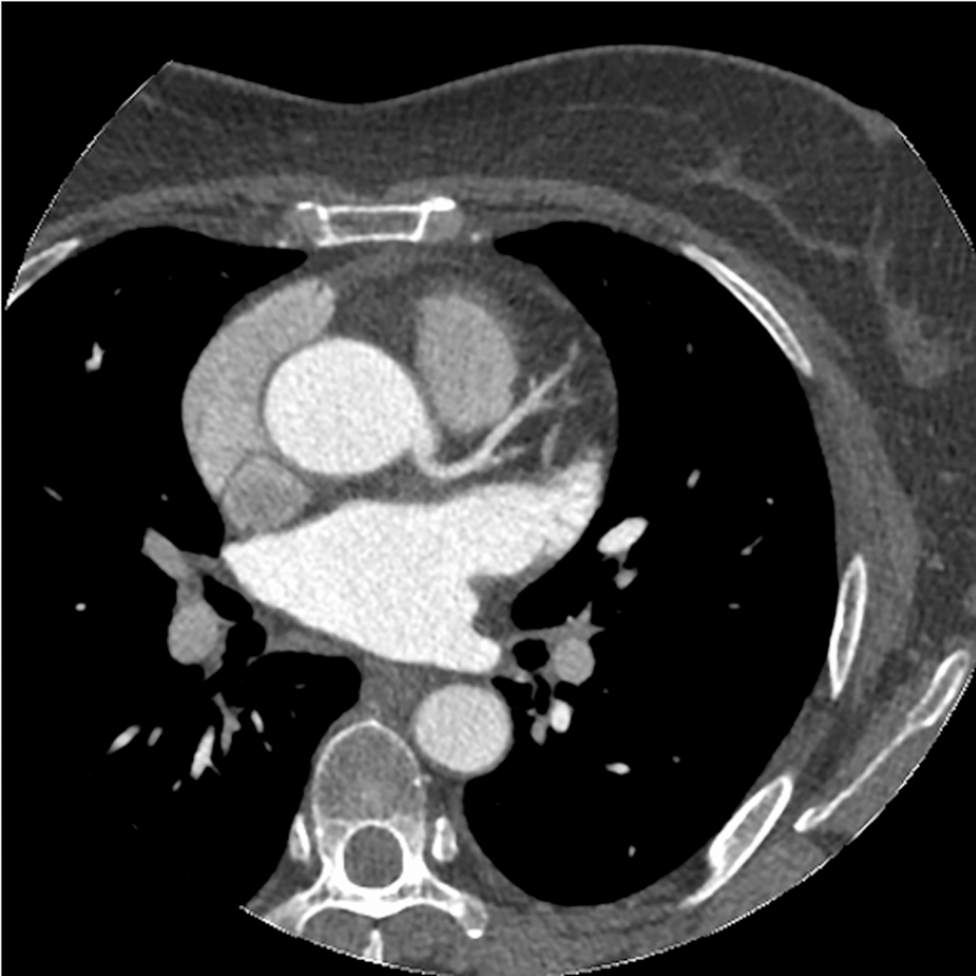
The axial image of CCTA in subgroup B2. Fifty-two years old, male, 75kg. CT values in target vessels are as following: AO = 388.12HU, LMA = 359.05HU, LAD = 339.61HU, LCX = 323.09HU.

**Fig 7:**
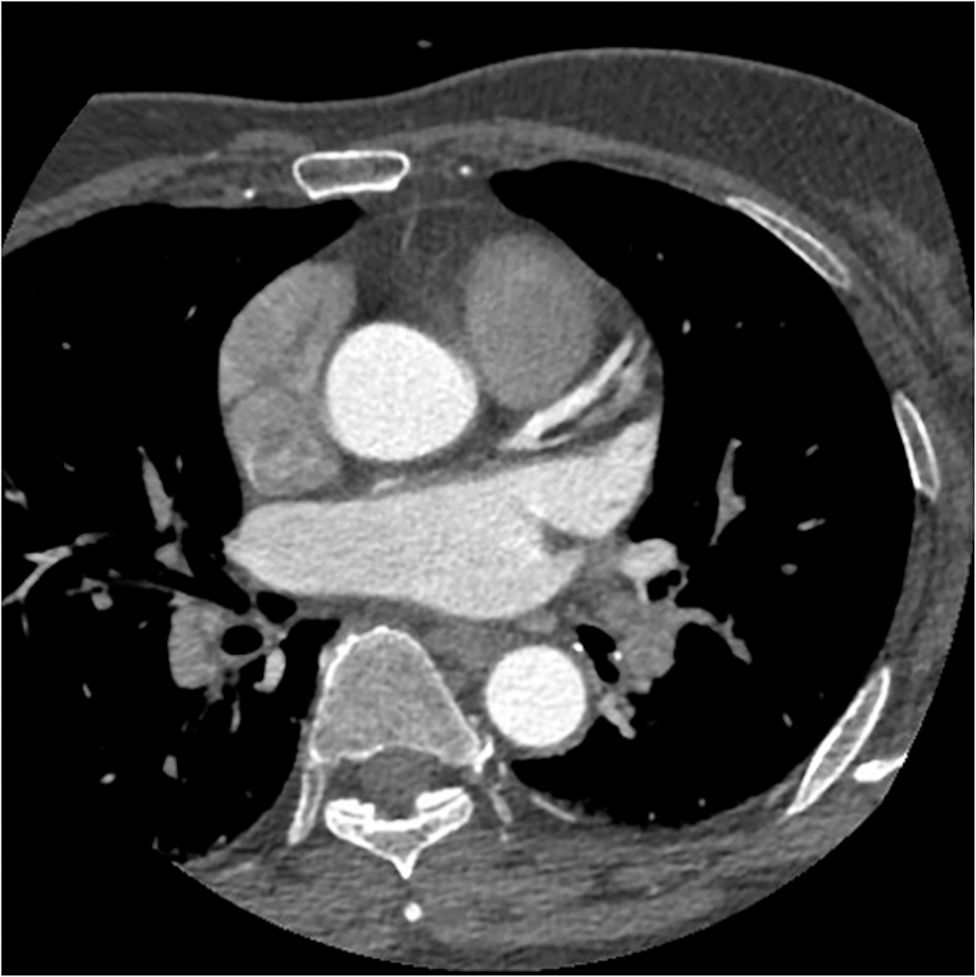
The axial image of CCTA in subgroup B3. Fifty-six years old, male,74kg. CT values in target vessels are as following: AO = 412.71HU, LMA = 380.02HU, LAD = 372.23HU, LCX = 349.72HU.

**Fig 8:**
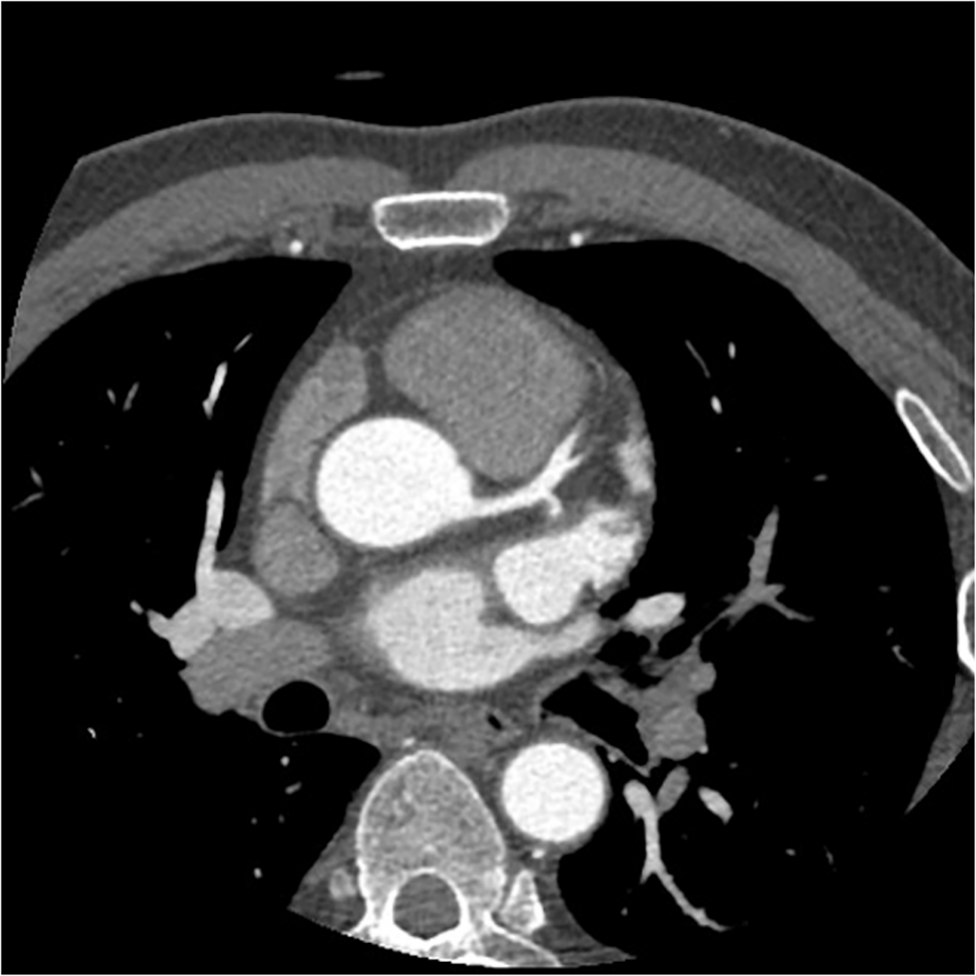
The axial image of CCTA in subgroup B4. Fifty-four years old, male, 75kg. CT values in target vessels areas following: AO = 473.45HU, LMA = 469.20HU, LAD = 455.17HU, LCX = 443.72HU.

For images in Group A, sufficient CT attenuation of between 317HU and 392HU was obtained for all target vessels with no difference among the 300 mg I/ml, 320 mg I/ml, 350 mg I/ml and 370 mg I/ml CM subgroups(Table 2)(Figs 1–4). For images in group B, except LMA and LAD, enhancements in the other coronary arteries(AO, LCX, RCA, LV and RCA-PDA) had significant differences. The magnitude of enhancement decreased proportionally with the decrease of contrast medium concentration(Table 3)(Figs 5–8).The inter-group pairwise comparison by LSD method showed the following findings: The statistical differences in the enhanced CT attenuation in target vessels were observed between subgroup B1 and subgroup B4, of which the statistical differences in the enhanced CT attenuation of AO, RCA, LV and RCA-PDA were all significant. There were statistical differences in the enhanced CT attenuation of AO, LV and RCA-PDA between subgroup B1 and subgroup B3. The statistical differences in the enhanced CT attenuation of RCA and RCA-PD were also found between subgroup B1 and subgroup B2. However, no statistically significant differences in the enhanced CT attenuation in target vessels were observed between other groups.

**Table 2:** Comparison of the enhancement degree in target vessels among sub-groups with different concentrations of CM in group A.

Sub- group	*n*	AO	LMA	LAD	LCX	RCA	LV	RCA- PDA
**A1**	20	385.0±32.93	359.37±34.42	361.77±36.80	355.38±39.63	350.17±39.51	359.11±47.16	325.86±51.94
**A2**	20	386.71±53.6	372.26±50.19	355.29±53.04	346.29±57.20	355.64±56.54	364.14±60.22	344.31±73.65
**A3**	20	392.03±43.33	368.29±49.91	370.04±36.45	355.14±38.67	352.82±46.39	342.26±55.84	317.11±69.15
**A4**	20	391.03±48.82	372.95±48.21	375.48±54.06	365.13±63.32	356.30±45.79	361.59±42.63	340.48±71.25
***F***		0.111	0.366	0.754	0.458	0.070	0.726	0.716
***P***		0.953	0.778	0.523	0.712	0.976	0.540	0.545

**Table 3:** Comparison of the enhancement degree in target vessels among sub-groups with different concentrations of contrast medium in group B.

Sub- group	*n*	AO	LMA	LAD	LCX	RCA	LV	RCA- PDA
**B1**	20	322.63±63.35	309.15±69.07	289.43±68.56	276.64±69.16	304.78±71.52	294.02±62.33	250.84±66.49
**B2**	20	359.92±59.31	345.12±59.73	321.36±65.63	336.40±66.35	345.29±78.81	348.39±71.75	327.94±75.43
**B3**	20	368.53±66.49	353.14±74.08	309.11±84.88	315.45±75.65	352.82±46.39	352.25±76.41	347.80±107.57
**B4**	20	402.37±82.32	370.98±88.78	346.78±76.21	343.67±76.50	369.11±78.28	356.70±78.13	315.09±84.37
***F***		4.574	2.489	2.092	3.487	3.093	3.298	4.844
***P***		0.005	0.067	0.108	0.020	0.032	0.025	0.004

**Figs 5–8. The axial images of CCTA in subgroups B.** Constant injection speed of 5.5 ml/s and injection volume of 80 ml were used in these subgroups.

### Comparison of coefficient of variation (CV) of the CT attenuation value in target vessels with the individualized-protocol and fixed-protocol at different concentrations.

**Fig 9:**
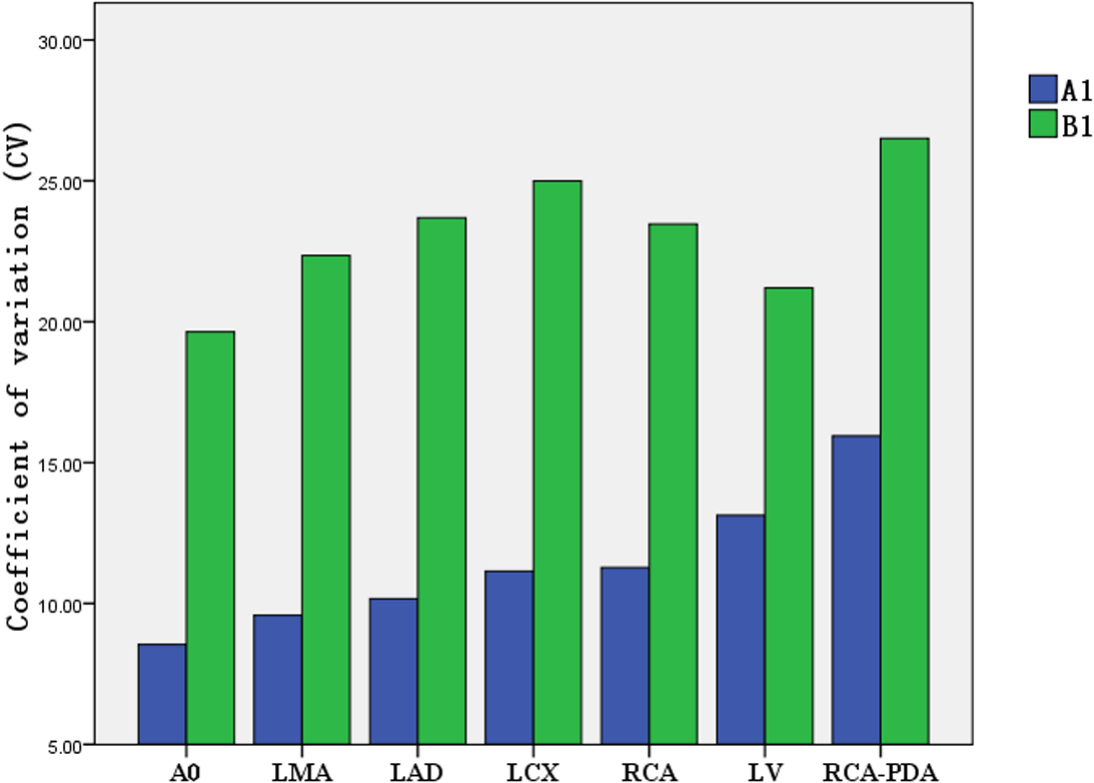
Comparison of CV for CT attenuation value between group A1/B1(300 mg I/ml).

**Fig 10:**
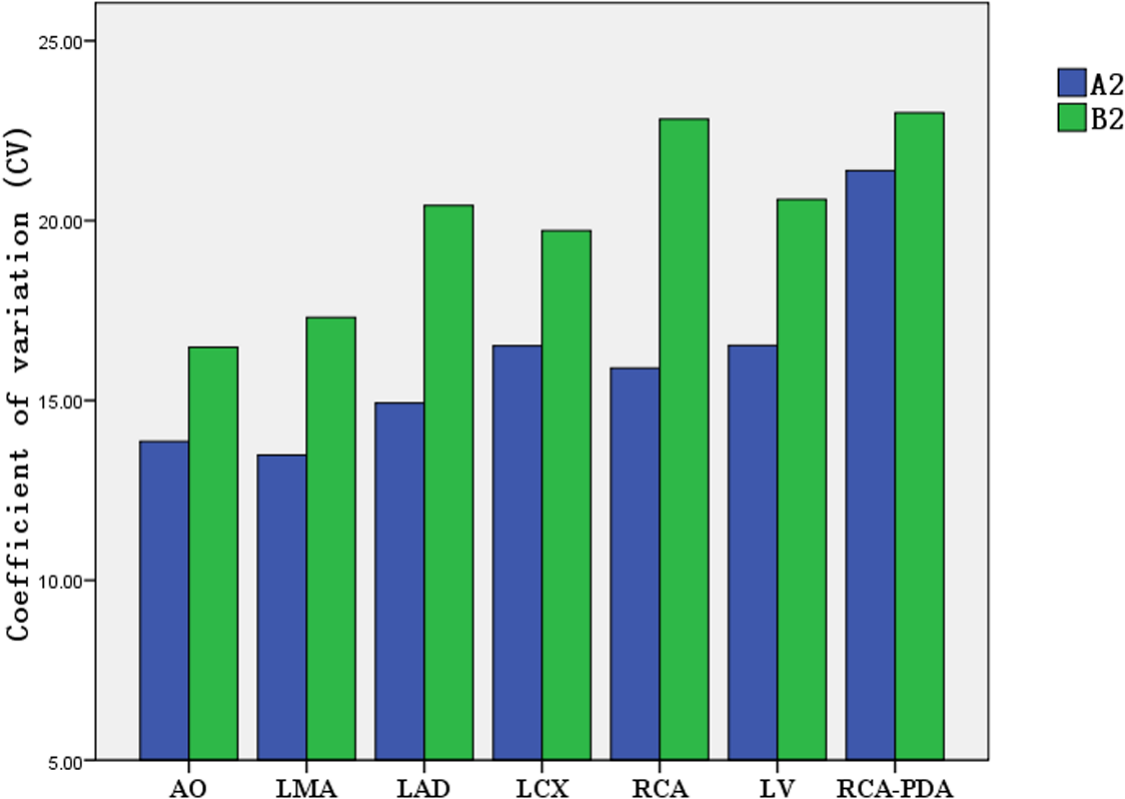
Comparison of CV for CT attenuation value between group A2/B2 (320 mg I/ml).

**Fig 11:**
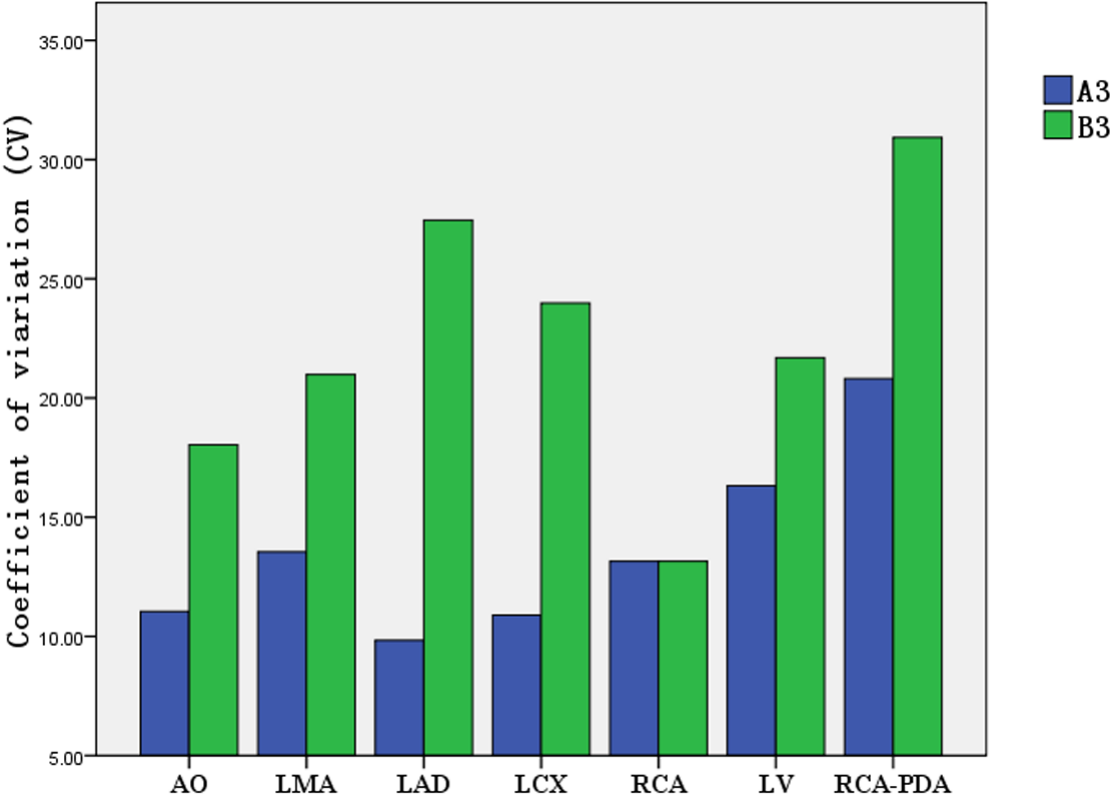
Comparison of CV for CT attenuation value between group A3/B3 (350 mg I/ml).

**Fig 12:**
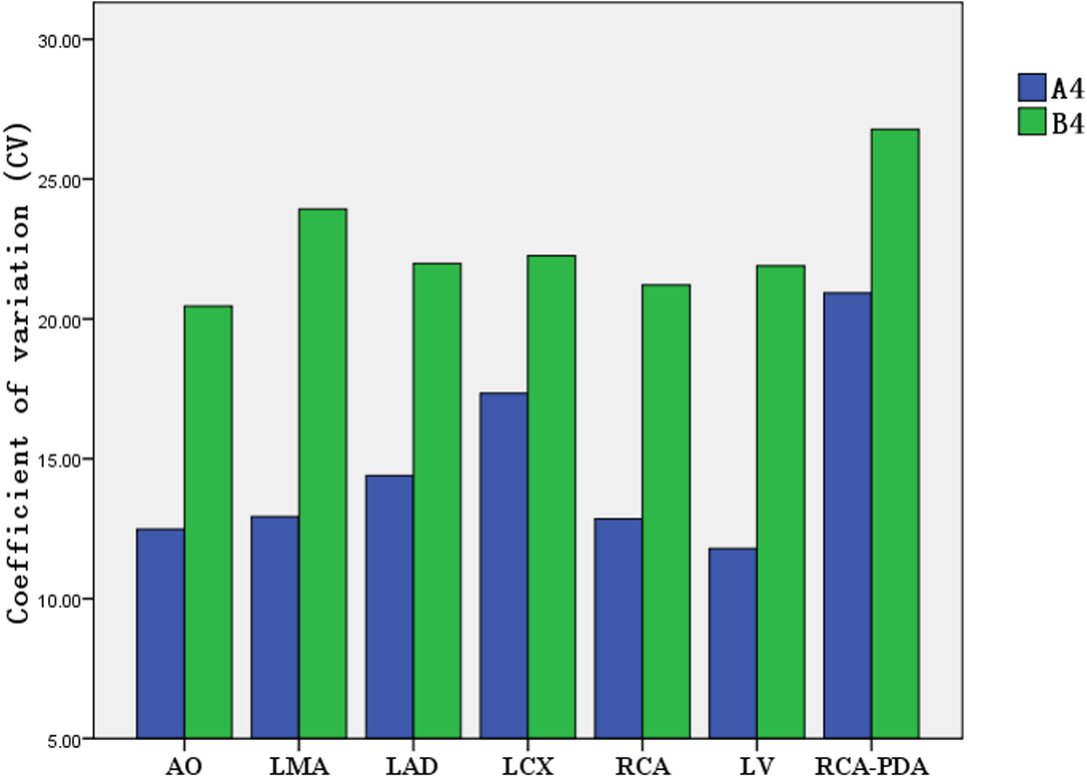
Comparison of CV for CT attenuation value between group A4/B4 (370 mg I/ml).

At each contrast medium concentration, CV of the CT attenuation value in target vessels in group A was lower than that in group B (Figs 9–12).

### Comparison of vessel enhancement degree of coronary arteries among different body weight subgroups in the individualized-protocol group

Eighty patients under group A were divided into 5 subgroups based on body weight: <60 kg, 60–69 kg, 70–79 kg, 80–89 kg and ≥90 kg. The comparison of vessel enhancement degree in target vessels among the different body weight subgroups showed no statistical difference (Table 4).

**Table 4:** Comparison of vessel enhancement degree of coronary arteries among sub-groups with different body weights in group A.

Body weight	n	AO	LMA	LAD	LCX	RCA	LV	RCA- PDA
**<60**	7	380.41±27.58	363.87±32.89	352.31±48.15	352.61±29.26	344.83±51.44	359.01±44.99	324.89±58.83
**60–69**	20	380.61±40.12	358.92±38.27	355.91±36.28	336.35±40.89	353.38±42.66	340.91±54.97	312.32±61.87
**70–79**	19	391.74±42.40	369.40±48.55	364.45±31.68	357.93±46.86	352.95±53.63	357.93±47.61	334.60±67.14
**80–89**	23	401.75±56.42	384.06±50.99	383.96±61.06	372.67±52.25	361.31±46.99	371.51±58.99	356.79±79.36
**≥90**	11	388.55±59.09	368.09±58.57	372.73±58.06	363.54±78.99	359.87±61.06	357.83±46.18	334.67±63.81
**F**		0.615	0.813		1.395	0.199	0.904	1.161
**χ2**				*4*.*636*				
**P**		0.654	0.521	0.327	0.244	0.938	0.466	0.335

Note:: The inclined data in this table had heterogeneity of variance and were analyzed with Kruskal-Wallis H test.

### Comparison of effective radiation dose, total contrast medium dose and flow rate under the individualized-protocol and conventional-protocol.

The average effective radiation dose had no significant difference between group A(11.34±2.99mSv) and group B(12.93±5.40 mSv)(*Z* = 1.720, *P* = 0.085). The average total contrast medium dose and average flow rate in group A were (62.96±8.25) ml and (5.24±8.47) ml/s, both lower than the respective value of 80 ml and 5.50 ml/sin group B (all *P* < 0.001).

## Discussion

Coronary arteries have small diameters and are tortuous, and CCTA image quality is critical for diagnostic accuracy, while reasonable use of CM is one of the major factors that influence imaging quality. An appropriate CM enhancement level is beneficial for the visualization of coronary arteries, over enhancement level may affect the visualization of calcified plaques[7]; and insufficient enhancement level may affect visualization of coronary arteries. However, the underestimation of non-calcified plaque area and then lumen stenosis may occur because of partial volume effect[8]. The enhancement degree of coronary arteries is positively correlated with the content of iodine in the contrast medium[9]. The enhancement in coronary arteries can be increased by elevating the flow rate of CM or using a higher concentration of CM[10]. Another important factor affects the level of enhancement is the patient weight. However, in clinical practice, the CM injection protocol with a constant speed and volume is widely used in clinic, and under such a protocol the individual variation among subjects including different concentrations of contrast medium, at different ages, and with different body types and different heart functions is ignored, which lead to discrepancy of enhancement degree among different subjects. This is not beneficial to visualize coronary arteries and coronary atherosclerotic plaques and thus reduces image quality and diagnostic quality[11].With the purpose of standardizing the use of CM during CCTA to achieve a similar enhancement effect of coronary arteries independent of CM concentration and patient weight, we proposed the individualized CM injection protocol. In this individualized CM protocol, the total volume of contrast medium was not only dependent on CM concentration, but also on patient weight, and in the end the total iodine load of 280 mgI/kg was obtained. We have evaluated this individualized CM protocol with patients of different weights and different CM concentrations to demonstrate the effectiveness of achieving more consistent CT attenuation. The enhancement is also affected by many the other factors such as blood volume, cardiac output, and scan time and tube voltage [3]. Sakaiet et al[12]demonstrated that the peak scan time obtained by low-dose pre-injection is closely related with heart function. In the present study, the same scanning parameters were used for the two groups, the delayed time was determined by a low-dose pre-injection method, and the effects of blood volume and cardiac output were balanced as much as possible, thus the data of two groups were comparable.

Under the individualized-protocol, the average enhanced CT attenuation in target vessels in the 300 mg I/ml, 320 mg I/ml, 350 mg I/ml and 370 mg I/ml CM groups was between 317HU and 392HU, and no statistical difference was observed among these groups. The study results of Fei et al suggested that with 64-slice CT, the optimal enhanced CT attenuation of coronary arteries for diagnosis of coronary stenosis was 350HU[8]. Therefore, the individualized-protocol for different CM concentrations and different body-weight individuals in this study can meet the requirements of clinical diagnosis. Under the fixed iodine-dose protocol, the average enhanced CT attenuation of LAD, LCX and RCA-PDA in the 300 mg I/ml CM group was <300HU. Among different concentrations of CM, there were statistical differences in the enhanced CT attenuation of AO, LCX, RCA, LV and RCA-PDA. It indicated that under the conventional fixed-protocol, the enhanced CT attenuation of coronary arteries was affected by CM concentration. In this study, the enhancement amplitude in target vessels with 370 mg I/ml CM was higher than that with 300 mg I/ml CM, which was consistent with the study results obtained by Cademartiri et al [13] via 16-slice CT with a CM injection protocol with a constant speed and volume (4.0 ml/s and 140 ml).

CV is an absolute value reflecting the discrete degree of data and measures the difference in the CT attenuation enhancement in target vessels between the weight-adjusted iodine-dose protocol group and the fixed contrast volume protocol group. In the present study, the lowest CV was observed for aortic opening in the 300 mg I/ml CM group under the individualized-protocol, at 8.55% for CT attenuation of different patients; the highest CV was observed for LAD in the 350 mg I/ml CM group under the conventional fixed-protocol, at 27.46%. In addition to the 350 mg I/ml CM group, CV for RCA under both the individualized-protocol and conventional fixed-protocol was 13.15%; in other CM groups, the CV values for coronary arteries, aortic opening and left ventricle were lower under the individualized-protocol than under the conventional fixed-protocol. This suggested that the homogeneity of enhancement effect between patients under the individualized-protocol was better.

In CCTA, the CT attenuation enhancement in coronary arteries is associated with many factors including body weight. The patients under the individualized-protocol were divided into 5 groups based on body weight for further analysis:<60 kg, 60–69 kg, 70–79 kg, 80–89 kg and ≥90 kg. No statistical difference in the enhancement degree in target vessels was found between different body weight groups, which indicated that the CM enhancement scan protocol tailored to body weight in this study was not influenced by body weight.

The average total contrast medium dose and flow rate in the individualized-protocol group were 63 ml and 5.2 ml/s, lower than the respective value in the conventional fixed-protocol group (80 ml and 5.5 ml/s). At present, most contrast media used are non-ionic and have high safety, but the iodine contrast medium is certainly toxic to renal cells and even cause contrast-induced nephropathy (CIN)[14]. The occurrence of CIN is positively correlated with the total dose of CM[15], thus the body weight-tailored CM injection protocol may reduce the occurrence of CIN. Our study did have some limitations. First, this investigation reflects our preliminary experience with a small number of patients, further studies with more patients, especially in the different weight subgroups, are needed to validate our results. Second, CT attenuation measurement in target vessels was the focus of this study, and we did not evaluate the general image quality or the diagnostic accuracy in comparison with the invasive coronary angiography. Finally, our scan protocol was limited to 120kVp tube voltage. The impact of using different tube voltages on our conclusions needs to be further studied.

To summarize, the individualized-protocol based on patient weight and contrast concentration provides overall contrast dose reduction and achieves more homogenous attenuation among different coronary vessels and patients, compared with the conventional contrast medium injection protocol with fixed volume and injection rate.

## Supporting Information

S1 Dataset (XLSX)

S2 Dataset (XLSX)

S3 Dataset (XLSX)

S4 Dataset (XLSX)
